# Vertical organic electrochemical transistor platforms for efficient electropolymerization of thiophene based oligomers†

**DOI:** 10.1039/d3tc04730j

**Published:** 2024-03-04

**Authors:** Maciej Gryszel, Donghak Byun, Bernhard Burtscher, Tobias Abrahamsson, Jan Brodsky, Daniel Theodore Simon, Magnus Berggren, Eric Daniel Glowacki, Xenofon Strakosas, Mary Jocelyn Donahue

**Affiliations:** a Laboratory of Organic Electronics, Department of Science and Technology, Linköping University 60174 Norrköping Sweden xenofon.strakosas@liu.se mary.donahue@liu.se; b Bioelectronics Materials and Devices Lab, Central European Institute of Technology, Brno University of Technology Purkyňova 123 61200 Brno Czech Republic; c Wallenberg Wood Science Center, Department of Science and Technology, Linköping University 60174 Norrköping Sweden

## Abstract

Organic electrochemical transistors (OECTs) have emerged as promising candidates for various fields, including bioelectronics, neuromorphic computing, biosensors, and wearable electronics. OECTs operate in aqueous solutions, exhibit high amplification properties, and offer ion-to-electron signal transduction. The OECT channel consists of a conducting polymer, with PEDOT:PSS receiving the most attention to date. While PEDOT:PSS is highly conductive, and benefits from optimized protocols using secondary dopants and detergents, new p-type and n-type polymers are emerging with desirable material properties. Among these, low-oxidation potential oligomers are highly enabling for bioelectronics applications, however the polymers resulting from their polymerization lag far behind in conductivity compared with the established PEDOT:PSS. In this work we show that by careful design of the OECT geometrical characteristics, we can overcome this limitation and achieve devices that are on-par with transistors employing PEDOT:PSS. We demonstrate that the vertical architecture allows for facile electropolymerization of a family of trimers that are polymerized in very low oxidation potentials, without the need for harsh chemicals or secondary dopants. Vertical and planar OECTs are compared using various characterization methods. We show that vOECTs are superior platforms in general and propose that the vertical architecture can be expanded for the realization of OECTs for various applications.

## Abstract 2

Vertical organic electrochemical transistor platforms enable facile channel formation by electropolymerization. The improved deposition control and resulting high performance is demonstrated here with the trimer ETE-COONa.

## Graphical abstract

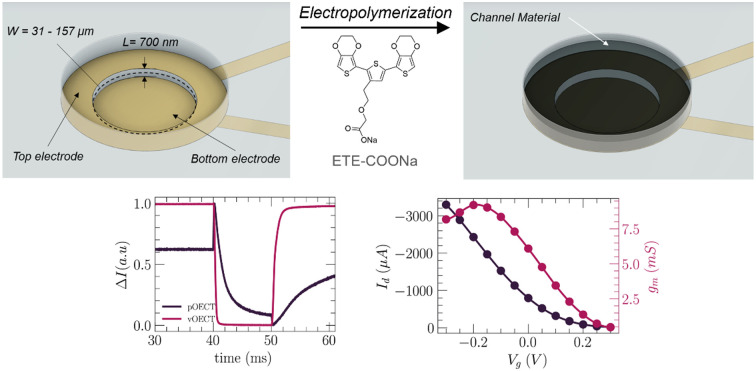

## Introduction

Advances in organic electrochemical transistors (OECTs) have provided new opportunities for the fields of bioelectronics, biosensors, and neuromorphics.^1–6^ This advancement is due in part to the development of new OECT channel materials with mixed ionic–electronic conduction, beneficial for interfacing directly with electrolyte environments.^7–13^ The mixed conduction mechanism of the semiconducting channel material is ideal for bioelectronic applications aiming to achieve signal transduction between ionic fluctuations and electrical currents. Although the OECT materials toolbox has greatly expanded over the past years, the practical need of solution processability to enable standard deposition techniques has limited the use of promising materials requiring electropolymerization. When using electropolymerization, planar OECT (pOECT) configurations typically result in non-uniform, bulky channels in order to bridge the source and drain contacts.^14^ The vertical OECT (vOECT) has been introduced as a variation on its planar counterpart, providing a useful geometry for facile reduction of the channel length, *L*, and additionally offering an advantageous platform for controlled electropolymerization.^15–19^ This provides an opportunity to greatly expand the materials practically used in OECTs.

**Fig. 1:**
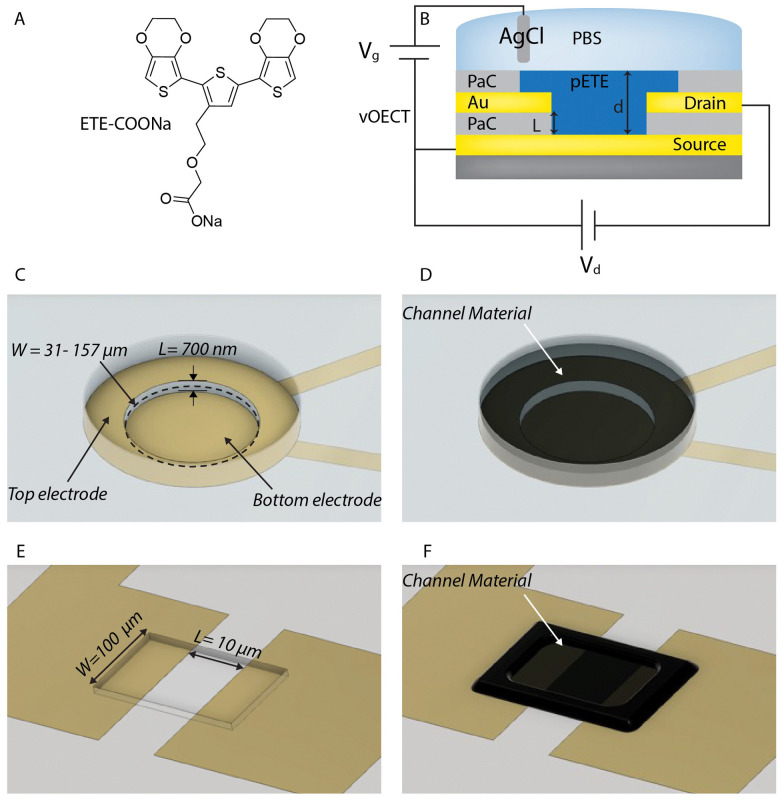
Electropolymerization of ETE-COONa with the vertical OECT architecture. (A) Schematic of the ETE trimer with a sodium 2-(2-ethoxy)acetate salt side chain (ETE-COONa) used as the channel material. (B) Vertical OECT cross sectional view, (C) angled top view without polymer channel showing utilized dimension of *L* = 700 nm, top contact opening of *Ø* = 10 μm, 20 μm, 50 μm giving *W* = 31 μm, 63 μm, 157 μm, and (D) angled top view with channel material. The circular channel opening provides a large channel width, *W*, while the vertical structure allows for reduction of the channel length, *L*. Together these factors increase the *W*/*L* ratio and thus the amplification of the transistor. (E) Angled top view (without polymer channel) of the pOECT channel, used for deposition and performance comparison with *L* = 10 μm and *W* = 100 μm and (F) angled top view with polymer channel of the pOECT channel.

Among recently developed promising materials, trimers based on a 2,5-bis(2,3-dihydrothieno[3,4-*b*][1,4]dioxin-5-yl)thiophene (ETE) (Fig. 1A) backbone have emerged as an interesting option.^20^ ETE trimers have a low oxidation potential, which has rendered them useful for neuromorphic applications^21,22^ and allows for oxidation by enzymes to form radicals.^23^ These radicals spontaneously polymerize forming conducting polymers. Based on this concept, *in vivo* polymerized electrodes have been formed in rose plants^24^ and in hydra.^25^ More recently enzymatically polymerized electrodes were formed in the zebrafish brain^26^ and in genetically modified mice brains.^27^ Compared to fine-tuned PEDOT:PSS, with formulations often resulting in conductivities in the range of 1000 S cm^−1^ or higher,^28^ the ETE trimer materials have relatively low conductivities on the order of 10 S cm^−1^. For applications in which high transconductance OECTs are desired to achieve high amplification, this conductivity range can be a limiting factor. Owing to their ease of fabrication and the small channel lengths, vertical transistors can compensate the low conductivity and improve the transistor performance.

In this work we compare vOECTs and pOECTs, in particular with regard to channel electropolymerization of ETE-COONa. We observe that the vOECT design is a better platform for electropolymerized channels, while also providing an ideal device structure for materials with intermediate conductivity. Whereas approaches such as substrate surface functionalization have been needed in the past to achieve polymerization across the pOECT channel gap,^21^ the vOECT geometry enables controlled electropolymerization and provides devices with improved performance and a smaller footprint when comparing to pOECTs. Formation of the channel, namely connection between source and drain electrodes, occurs faster and requires less material. Making use of this efficiency, we demonstrate that vOECTs have higher currents, transconductance, and faster response, while less polymerization time and trimer quantity is needed for channel formation.

## Results

vOECTs and pOECTs were compared to investigate electropolymerization efficiency and OECT performance of the trimer ETE-COONa as the channel material. In order to maximize performance in general, and in particular for ETE-COONa with modest conductivity, the vOECT structure was targeted to provide a short channel length, *L*, and a beneficial architecture for channel deposition through electropolymerization (Fig. 1). The definition of *L* by the thickness of the insulation layer between source and drain electrodes allows for a straightforward means to achieve high amplification by increasing the transistor width (*W*) to length ratio (*W*/*L*). Additionally, the reduced gap between electrodes and the vertical orientation facilitates fast channel formation by electropolymerization, overcoming the typical deposition challenges for electropolymerized OECT channels. Photolithography methods were employed to create both vOECTs and pOECTs. While pOECTs were fabricated using a single metal patterning step and a subsequent parylene C (PaC) insulation layer,^29^ vOECTs consisted of two layers of patterned Au lines to be used as source and drain contacts, separated by a thin PaC layer of approximately 700 nm. A second PaC layer acts as the upper insulation layer. A circular geometry of the upper vOECT contact is used to allow for a high *W*/*L* ratio in a compact structure with the channel *W* defined by the circumference of the opening (Fig. 1C). The circular design additionally allows for a uniform deposition (Fig. 1D). The channel dimensions and design of the pOECT used for comparison is portrayed in Fig. 1E and F.

### Electropolymerization of the OECT channel

**Fig. 2:**
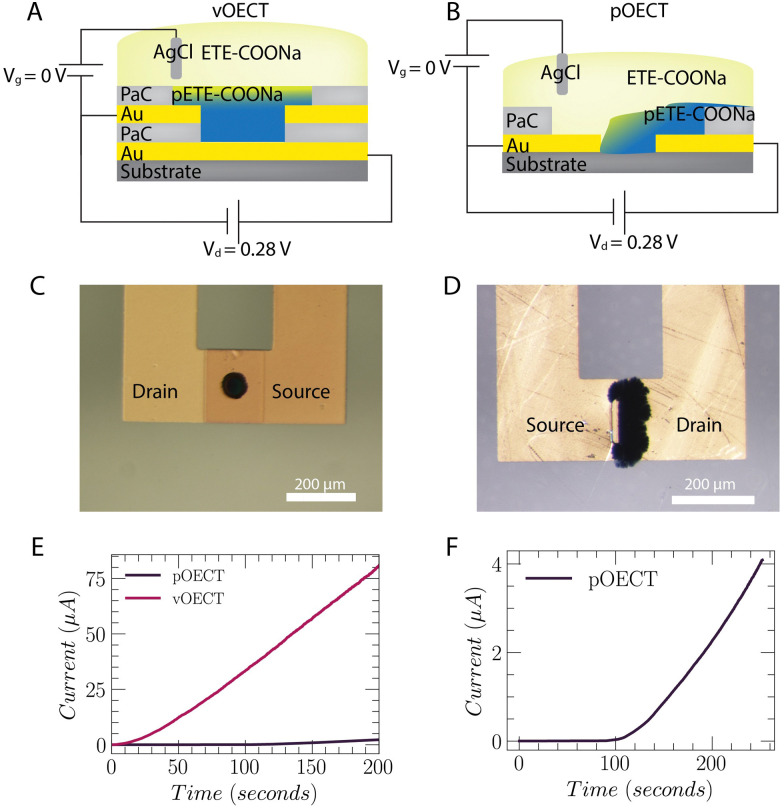
vOECT *versus* pOECT channel electropolymerization. By applying the electropolymerization potential to a single electrode contact with respect to the shorted second contact and Ag/AgCl pellet, the current, *I*_DS_, may be monitored during channel growth. (A) and (B) Schematic of electrodeposition set-up for trimers in the vertical vs planar configuration. When using the vertical architecture the electropolymerization potential is applied to the bottom electrode contact while when depositing on the planar structure a single contact is used (here this is depicted as the polymerization potential applied to the right-hand contact). (C) Optical image showing the resulting vOECT. The dimensions of the Au pattern are *W* = 157 μm (*Ø* = 50 μm), *L* = 700 nm. (D) Optical image showing the resulting pOECT. The dimensions of the Au pattern are *W* = 100 μm, *L* = 10 μm. (E) and (F) Current between source and drain contacts during deposition when applying the +0.28 V polymerization potential, demonstrating significantly faster channel bridging (∼10 s seen in (E)) for the vOECT.

ETE-COONa dissolved in DI water was electrodeposited onto the substrates by three different methods, all applying a low positive voltage, *V* = 0.28 V. This electropolymerization potential was chosen after carrying out initial tests using galvanostatic and potentiostatic processes, *i.e.* with constant current or constant voltage. The monomer was sensitive to potentials higher than 0.3 V, resulting in non-uniform structures (see Fig. S1, ESI†). Thus, application of constant voltage of 0.28 V was employed to ensure that the polymerization potential remains below this value. To carry out the deposition, we first investigated Method 1 – application of the electropolymerization potential to the bottom electrode of the vOECT *versus* an Ag/AgCl pellet shorted to the top electrode (Fig. 2A). The Ag/AgCl pellet thus acts as an auxiliary electrode, mainly responsible for charge compensation during the electropolymerization. During the electropolymerization process, the trimers are oxidized at the positive drain electrode, forming radicals. The radicals further polymerize and stack to create aggregates. The polymer aggregates precipitate and deposit at the bottom electrode spatially augmenting its conductive area. As the polymer is created, its moving front reaches the upper electrode, bridging the contacts to form the transistor channel. Similarly, for the pOECT, the electropolymerization potential (0.28 V) was applied at a single planar contact (illustrated as the right-hand side contact in Fig. 2B). In Fig. 2C and D, microscope images resulting from deposition Method 1 (application of the potential to a single electrode) depict the formation of channels for both vOECT and pOECTs. Utilization of this method allows for electrical monitoring of the formation and evolution of the OECT channel. Upon formation of the channel, a source/drain current results (*I*_DS_) (Fig. 2E and F). Fig. 2E shows the evolution of the current over time during electropolymerization for both pOECTs and vOECTs. In vOECTs, the channel forms with an average time of 10 s. Post channel formation, the current increases in a linear fashion due to the continuous deposition of pETE-COONa, which alters the channel thickness. For pOECTs, the formation of the channel takes on average 10 times longer than vOECTs. In addition to the significantly faster time to bridge the source and drain in the case of the vOECT, the *I*_DS_ current also increases with a steeper slope after making the connection. This result implies that the rate of polymer increase, or the rate that the channel thickness changes, is more uniform in vOECTs compared to pOECTs. It is evident in Fig. 2C that the vOECT channel is uniformly distributed creating a homogeneous layer and facilitating better characterization of the geometrical characteristics. Since the polymer propagates from the bottom electrode, it is initially confined and grows in the vertical direction toward the other transistor channel contact. For pOECTs, the polymer may grow randomly in any direction, resulting in poor uniformity (Fig. 2D) and affecting reproducibility. Less electrodeposited material is therefore required for the formation of the channel in vOECTs compared to pOECTS. The difference in the amount of deposited material may be understood using the total polymerization charge, calculated from the current during the deposition process, as well as by observing the electrochemical impedance spectroscopy (EIS) and extracting the electrochemical capacitance values for each channel (refer to Table S1, ESI†). The channel growth benefits of the vOECT are a result of the circular design, and the small channel length.

We additionally investigated two alternative approaches for channel growth: Method 2 – electropolymerization using shorted contacts, and Method 3 – pulsed electropolymerization, alternating between applying the polymerization potential to each contact. In Method 2, the vOECT top and bottom contacts are shorted and the electropolymerization potential (+0.28 V) is applied *versus* the Ag/AgCl pellet (Fig. S2A, ESI†). Similarly, for the pOECT both planar contacts are shorted and channel polymerization is carried out (Fig. S2B, ESI†). In Method 3, for both vOECTs and pOECTs, the electropolymerization potential is applied for 5 seconds to each contact in alternation until the desired amount of total deposition time is achieved. For pOECTs, when Method 2 is used with the contacts shorted during electrodeposition, the film homogeneity is increased (Fig. S2D, ESI†). However, when using this method for both pOECT and vOECTs, there is a spread of the film towards the outside of the PaC insulator (Fig. S2C and D, ESI†), much more significant in the pOECT case. Analysis of the resulting channel morphology by scanning electron microscopy (SEM) provides a visual demonstration of the difference in polymer homogeneity for the two transistor geometries (Fig. S3 and S4, ESI†). The bulky material deposition for the pOECT is evident from the large channel volume (Fig. S3B and D, ESI†) and extreme outgrowth away from the patterned channel opening (Fig. S4, ESI†). Overall, the deposition method has a larger effect on the homogeneity in pOECTs, while the vOECT is particularly tolerant of variations in the deposition approach. Specific examples will be given in the following discussion regarding the transistor characteristics when varying the method for both pOECTs and vOECTs. Due to the design and geometry of the vOECT, the channel formation is homogeneous (Fig. 2C) and does not require special techniques such as surface functionalization.^30^ This allows for a more controllable *W*/*L* ratio, improving the characteristics of the devices and the batch-to-batch reproducibility.

### Electrochemical characterization

**Fig. 3:**
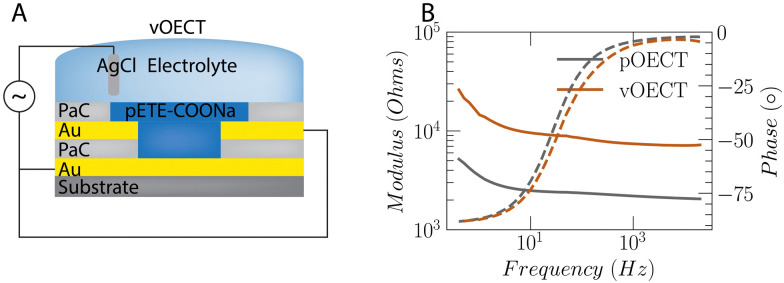
Electrochemical impedance spectroscopy of the electropolymerized OECT channel. (A) Connection schematic showing shorted contacts during the EIS characterization, depicted here for the vOECT architecture, analogous in the case of the pOECT with source and drain contacts shorted for application of the small signal (10 mV) *versus* the Ag/AgCl electrode. (B) Impedance data of polymerized channel grown using Method 1 – polymerization from the vOECT bottom contact or polymerization from a single pOECT contact. The larger amount of channel from the material for the pOECT *versus* the vOECT is evident by the lower electrochemical impedance, shown through bode plots – modulus (solid lines) and phase (dashed lines) – for pOECTs (gray) and vOECTs (orange line).

Although the current registered between source and drain contacts for pOECTs is smaller overall *versus* vOECTs during deposition, EIS measurements on the channel material show that the impedance of the pOECT channel is significantly lower than that of the vOECT (Fig. 3). This result indicates a larger volume of deposited material and a higher effective capacitance of the pOECT. As the specific capacitance of the trimers is about 25 F g^−1^,^26,31^ the effective capacitance is proportional to the deposited material. It is clear from Fig. 2D that in pOECTs, most of the polymer is deposited on the drain electrode. Moreover, depending on the surface properties of the insulator, the polymer film may extend on top of the PaC insulator rather than crossing the channel gap. This is prominent when the surrounding insulation surface is more hydrophobic than the channel substrate. Priming the surface between pOECT source and drain contacts with hydrophobic monolayers may improve the homogeneity of the channel, however, this necessitates additional surface functionalization steps^21^ or implementation of different deposition protocols that allow for more uniform deposition as discussed above using Method 2. When examining the impedance, the vOECT exhibits higher values as it necessitates less polymer for the formation of the channel than the pOECT (Fig. S5, ESI†). An equivalent circuit of a constant phase element (CPE) in parallel with a resistor and capacitor best fits the EIS data (Fig. S6, ESI†). The CPE corresponds to the polymer capacitor, the parallel resistor is used for inhomogeneities in the polymer microstructure, and the *C*_el_ capacitor corresponds to the electrode with contributions from both the gold and polymer.^32^ The value of *C*_el_ obtained from our fitting shows that these components cannot not be extracted separately. A series resistance is used to fit the electrolyte resistance. This model can be utilized when the films are thick or inhomogeneous.

### OECT performance

**Fig. 4:**
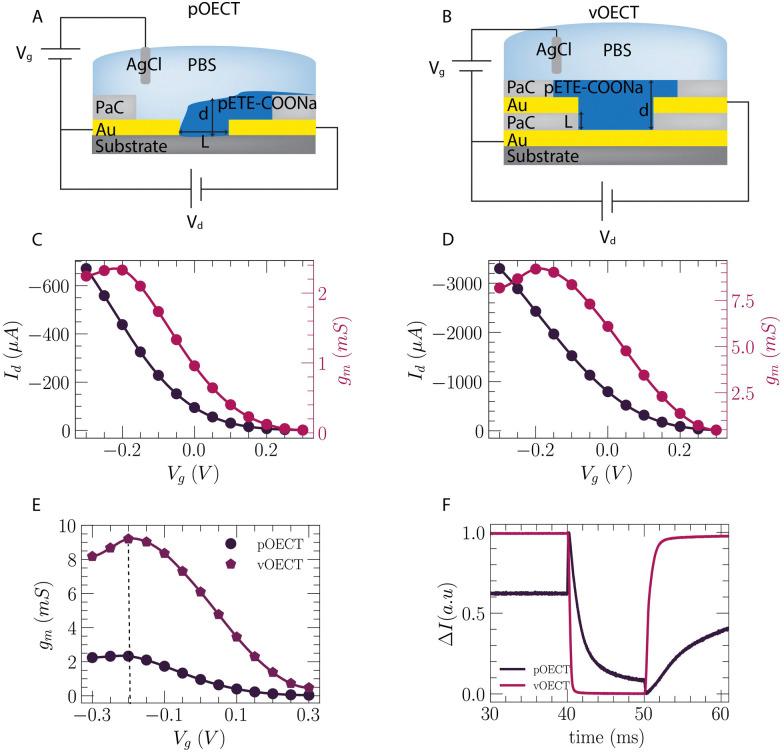
Planar and vertical OECT characteristics. Circuit schematic for measurement of pOECT (A) and vOECT (B). Transfer curve (black, *V*_D_ = −0.5 V) and derived transconductance (red), *g*_m_, of a pOECT with *W* = 100 μm, *L* = 10 μm (C) and vOECT with *W* = 157 μm (*Ø* = 50 μm), *L* = 700 nm (D) for channels formed by Method 1. (E) Transconductance comparison for pOECT (black) and vOECT (magenta) from (C) and (D). (F) Comparison of response time for pOECT and vOECT produced by Method 1.

Both pOECTs and vOECTs were connected as three-terminal devices to study their transistor characteristics (Fig. 4A and B). Fig. 4C and D show the transfer characteristics of the pOECT and vOECT, respectively, shown in Fig. 2, with the channel grown by Method 1. The source–drain current, *I*_DS_, is plotted *versus* the gate voltage, *V*_G_ (dark line), along with the transconductance (red line), *g*_m_ = Δ*I*_DS_/Δ*V*_GS_, which is the slope of the transfer curve. Although the large amount of deposited polymer observed for the pOECT in Fig. 2D may lead to the expectation that the attainable current will be higher, its peak current is in the sub-mA range, and the peak transconductance is 2 mS (Fig. 4C) (width-normalized pOECT peak transconductance: *g*_m_/*W* = 23.2 S m^−1^), while the peak current for vOECTs is in the mA range and its transconductance peak is ∼8 mS (Fig. 4D, vOECT: *g*_m_/*W* = 58.6 S m^−1^). Both planar and vertical architectures show peak transconductance in the negative gate voltage regime, at approximately *V*_G_ = −0.2 V, with the vOECT demonstrating values ∼5× higher when compared to the pOECT (Fig. S7 and S8, ESI†). The peak *V*_g_ at negative voltage is attributed to the pETE-COONa channel material which is not fully doped in the deposited state,^30^ but is further doped when a negative gate bias is applied. Despite the higher current and transconductance of the vOECT, often a result of larger volumes of polymer which in turn results in slower devices,^33^ its ON/OFF speed is much faster when compared to the pOECT (Fig. 4F). This is crucial for applications in which fast response time is important, in particular biological applications benefitting from the mixed ionic–electronic conduction of the OECT such as measuring high frequency brain activity.^34^ Representative time constant values for the vOECT are: *t*_off_ = 160 μs ± 1.1 μs, *t*_on_ = 1 ms ± 6.4 μs, while the pOECT demonstrates *t*_off_ = 3.44 ms ± 20 μs, *t*_on_ = 16 ms ± 20 μs (time constants are extracted as shown in Fig. S9, ESI†). It should be noted that the pOECT time constants were extracted from Fig S9 (ESI†) and not the data shown in Fig. 4F as the 10 μs pulse duration is not long enough to obtain correct values (Fig. S9B, ESI†). These findings highlight the benefit of the vertical geometry, namely faster channel formation, more reproducible electropolymerization, higher transconductance, and superior speed performance.

### Comparison of OECT performance for various deposition approaches

**Fig. 5:**
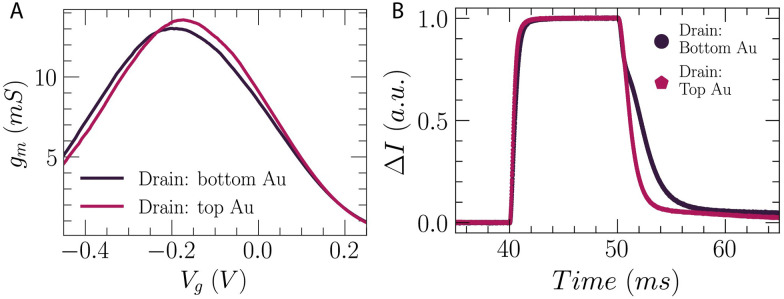
Optimization of polymerization time for transconductance and speed of vertical OECTs. (A) Transconductance shift when changing which contact (top or bottom electrode) is used as the OECT drain during characterization. *V*_D_ = −0.5 V (B) differences in transistor speed analyzed by pulse measurement^33^ when using top or bottom contact as the drain. Method 2: polymerization with shorted contacts. *V* = 0.28 V, 80 seconds.

Depending on the utilized channel deposition technique, the vOECT architecture, and the measurement set-up, one may control and fine-tune the resulting transistor characteristics. This control is possible through geometry variations which have been often investigated,^15,33^ but may also be affected by factors such as the drain/source connection scheme (Fig. S10, ESI†). This is particularly true if the polymer/electrode overlap and the overall channel are not perfectly symmetric. For example, when using the various methods described here for electrodeposition of the channel material on the vOECT, a different amount of polymer is deposited on the bottom or top electrode. In Method 1 this is a result of polymerization from the bottom contact only, which eventually overlaps onto the top contact, while Methods 2 and 3 result in different amounts of polymer because the surface area of the top and bottom contacts is not the same. When carrying out measurements on these types of channels, different results are observed depending on which electrode is set as the source or drain contact. The transconductance peak position and overall value may be different as well, as is seen in the example in Fig. 5A for a channel deposited by Method 2 with shorted contacts. When increasing the channel thickness, higher *g*_m_ values are attained as expected for larger polymer volumes, and a different peak *g*_m_ relationship is observed when comparing top or bottom drain connection (Fig. S11, ESI†). Apart from transconductance, the response time of the same channel can differ depending on the drain/source connection choice as well (Fig. 5B). This can be utilized as an added device benefit, namely, the same transistor can exhibit two different peak transconductance regimes and response time values. Although in this example the shift is relatively small, careful polymerization parameters and methods can yield higher differences. This may be useful, for example, to either increase the amplification when possible for slower biopotential recordings, or increase transistor speed for higher frequency activity when needed.^33^

An overall comparison of the transconductance shift depending on the deposition method and the diameter of the opening in the upper vOECT contact is given in Fig. S10 (ESI†). Since the opening in the upper contact opening defines the *W* of the OECT and therefore improves the *W*/*L* ratio with increasing diameter, the increase in amplification within a deposition method is expected. The exhibited differences in the peak *g*_m_ and the gap between peak *g*_m_ values when varying the drain contact connection scheme give an indication of results that are easily attainable. In general, the vOECT provides a reliable structure for channel electropolymerization independent of electropolymerization method and without the need of additional processes or functionalization steps. Where taking advantage of different amplification levels and transistor speeds within a single device is desired, the results can be further tuned to achieve useful parameters for a specific application. The values obtained in this study for 40 second deposition using each polymerization method are given in Table S2 (ESI†). Method 1 produces devices with the lowest transconductance for a given deposition time, but with the largest difference in peak *g*_m_ gap (percentage difference: Δ*g*_m_/*g*_m,peak_) and the biggest shift in peak *g*_m_ gate potential. Method 3 gives the highest *g*_m_ for a given deposition time, and Methods 2 and 3 are similar regarding shifts in *g*_m_ as a result of drain connection. Lastly, it is worth mentioning that after storing the devices in ambient conditions, no measurable channel current is typically seen for pOECTs indicating degradation of the channel. This effect is not observed for vOECTs and is likely a result of the sensitivity of the pOECT device being stored in the dry state, while the vOECT remains functional when exposed to analogous conditions.

## Conclusions

In this work, we explored vertical structures as platforms for efficient electropolymerization of channel materials for OECTs. To perform this investigation, we chose the organic trimer ETE-COONa as a challenging candidate based on moderate conductivity and known difficulties when attempting polymerization across planar channels. We compared the vOECTs with the planar equivalents and we show that the vertical configuration offers multiple benefits, including fast channel formation, higher transconductance, faster ON/OFF switching times, higher reproducibility, and stability. These advantages are amplified for conducting polymers of medium range conductivity. This work highlights the broad applicability and benefits of vertical structures for realizing high performance OECTs for organic electronics.

## Methods

Material synthesis and characterization of monomer ETE-COONa (sodium 2-(2-(2,5-bis(2,3-dihydrothieno[3,4-*b*][1,4]dioxin-5-yl)thiophen-3-yl)ethoxy)acetate) have previously been reported.^26^

### pOECT/vOECT fabrication

vOECTs were fabricated based on previously established methods.^15^ Glass microscope slides were cleaned by immersion and sonication in 2% Decon 90 soap solution, acetone, and isopropanol solutions. The first (lower) metal interconnections were photolithographically defined using a negative photoresist (AZ nLOF 2070), a SUSS MA6 mask aligner, and AZ 726 MIF developer. Chromium and gold layers (3 nm/80 nm thick) were thermally evaporated onto the substrate, following surface treatment with oxygen plasma (100 W, 300 s, Diener electronic GmbH) to activate and clean the surface, and the metal patterns were formed by life-off process. An intermediate insulating thin (∼700 nm) polymeric film, Parylene C (PaC), layer was deposited onto the substrate, by chemical vapor deposition (Diener electronic GmbH) with help of an adhesion promotor (A-174, methacryloxypropyl trimethoxysilane). To build a concentrically aligned upper electrode, a second metal patterns were formed on the PaC layer in a manner similar to that aforementioned. The substate was then encapsulated by depositing another 1 μm thick PaC layer. The PaC layers were etched by reactive ion etching (150 W, O_2_ 500 sccm/CF_4_ 100 sccm) to open electrodes and contact pads. For this, a positive photoresist (AZ 10XT) was used as an etch mask. As the contact pads have relatively larger area to be etched than that of source/drain electrodes, it is required to appropriately control the etching time to circumvent the collapse of the 2nd metalized layer, that might lead to a short circuit between source and drain electrodes, due to the over etching of the interlayer when creating openings. The former sites were etched first while the site of smaller and stacked electrodes was masked with a piece of polydimethylsiloxane (PDMS), and then other openings were made for the latter.

### Electropolymerization

The detailed protocol for the synthesis of ETE-COONa was adapted from previous work.^26^ For electrochemical polymerization, a solution of ETE-COONa in DI water (1 mg ml^−1^) was used without any additional electrolyte. Test electropolymerization with ETE-COONa of the same concertation in 0.1M KCl or 0.033M Na_2_SO_4_ resulted in transistors of lower performance. Electropolymerization was performed in 2 electrode system, using Keithley K2600A Source meter. Depending on the type of samples under fabrication, working electrode was either bottom, top or both (shorted) vOECT contacts, on which the polymer was electrochemically deposited with varying time constant potential +0.28 V *vs.* Ag/AgCl pellet counter electrode. Despite lack of Cl- ions in the solution taken for electropolymerization, the electrode can be still regarded as non-polarizable thanks to chloride ions release from Ag/AgCl upon reduction of AgCl to Ag^0^ and Cl^−^ driven by a flow of negative current. We also attempted polymerizations with constant current, however the reproducibility was low. In the initial experiments, +0.28 V potential turned out to be optimal, using a higher potential resulted in channels of poor performance and material distribution, while using lower potential requires longer polymerization time without any improvement in the transistor performance. To show simplicity of the method, OECT substrates were only washed with DI before deposition of polymer, neither plasma nor electrochemical cleaning of Au were used. Impedance was taken by using a Gamry 1010B potentiostat. The OECT characteristics were acquired by a Keithley 2612B and custom LabVIEW software. The fast time response was acquired by a NI PXI with SMU and custom LabVIEW software. The schematics were created using Blender 3d and adobe illustrator. The data analysis was performed by using python 3.9.

### Electrochemical impedance measurements

Electrochemical impedance measurements were done with an Ivium technologies Vertex One potentiostat using platinum plate as counter electrode and Ag/AgCl pseudoreference electrode confined in a syringe, filled with the electrolyte (1× PBS, pH 7.4) by adjusting the pressure with another syringe connected by a flexible tubing. The sample was precisely positioned under the syringe tip to achieve the electrolytic contact without any mechanical connection. The electrical connection (as working electrode) was carefully established with a needle probes placed on contact pads of both transistor terminals. Spectra were recorded using a single-sine probing AC voltage of 10 mV amplitude at a DC bias of 0 V *vs.* the reference electrode with 20 points per decade.

## Author contributions

Conceptualization: MJD, XS, methodology: XS, MG, DB, MJD, monomer synthesis: TA, device fabrication and electrical characterization: XS, MG, DB, BB, JB, supervision. MJD, XS, EDG, writing – original draft: XS, MJD, MG, writing – review and editing: all authors, resources: EDG, DTS, MB.

## Conflicts of interest

There are no conflicts to declare.

## Supplementary Material

TC-012-D3TC04730J-s001
