# Supplementary material for: Vertical organic electrochemical transistor platforms for efficient electropolymerization of thiophene based oligomers
Source: J Mater Chem C Mater. 2024 Mar 4;12(15):5339–46. doi: 10.1039/d3tc04730j (PMC11025323; doi:10.1039/d3tc04730j)
Supplement: TC-012-D3TC04730J-s001 [file TC-012-D3TC04730J-s001.pdf]

Electronic Supplementary Material (ESI) for Journal of Materials Chemistry C.
This journal is © The Royal Society of Chemistry 2024

Supplementary Information: Vertical Organic Electrochemical Transistor Platforms for Efficient Electropolymerization of Thiophene Based Oligomers

Maciej Gryszel,[†a] Donghak Byun,[†a] Bernhard Burtscher[1], Tobias Abrahamsson[1], Jan Brodsky[2], Daniel T. Simon[1], Magnus Berggren[1,3], Eric D. Glowacki[2], Xenofon Strakosas[1]*, Mary J. Donahue[1]*

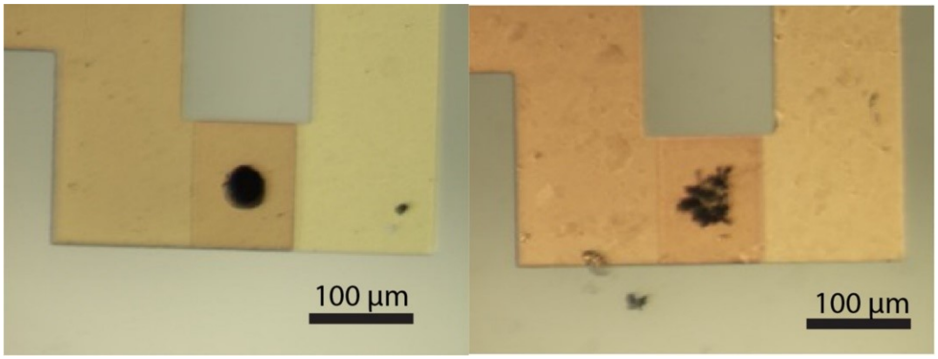

Figure S1: Microscope image showing the difference in resulting channel with relatively small changes in the applied polymerization potential (*left* 0.28 V, *right* 0.35 V).

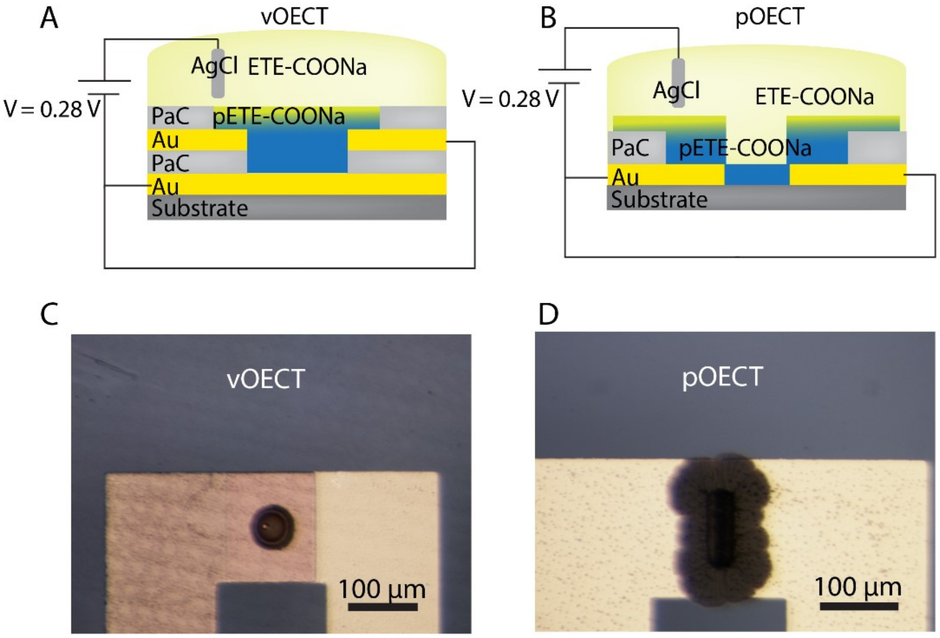

Figure S2: Schematics and microscope images of pETE-COOH OECT channel in which contacts were shorted during electropolymerization. Schematic of vOECT (A) and pOECT (B) in ETE-COONa water solution in which V = +0.28 V vs a Ag/AgCl pellet is applied to form a pETE-COOH OECT channel. C) Microscope image of a vOECT with 80 second polymerization time and Au pattern dimensions of W = 157 µm (Ø = 50 µm), L = 700 nm. D) Microscope image for a pOECT with channel formed by a polymerization time of 240 seconds. The dimensions of the pattern are W = 100 µm, L = 10 µm.

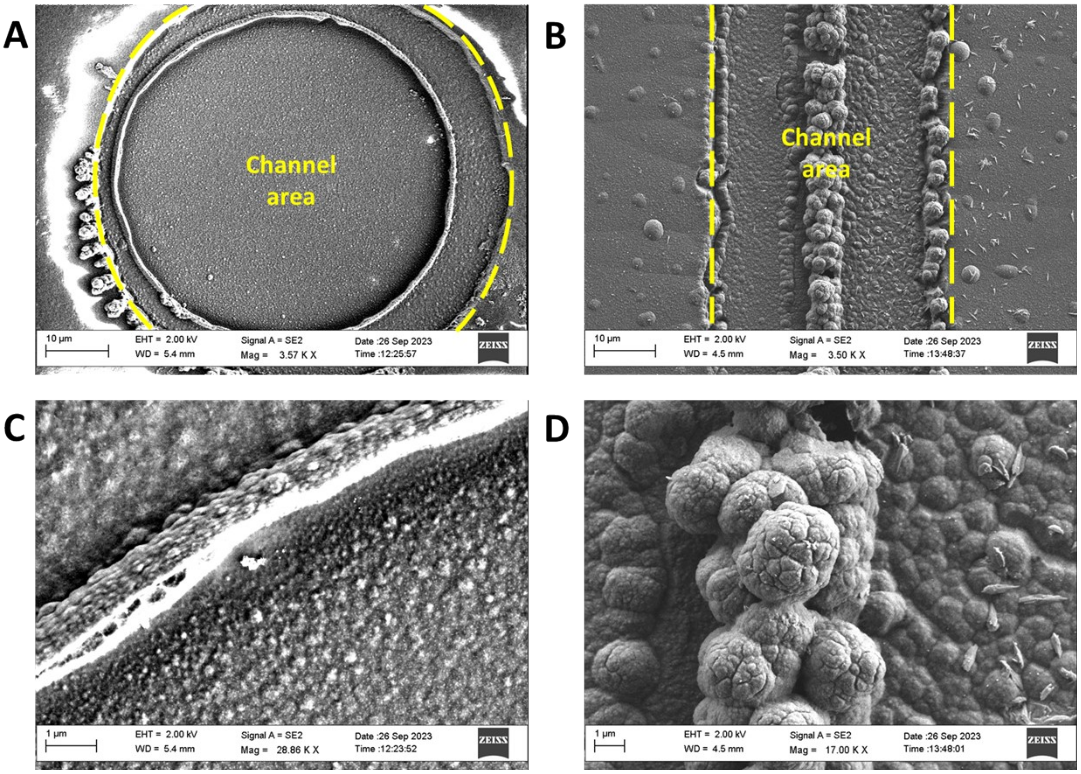

Figure S3: SEM comparison of vertical (A,C) and planar (B, D) channels after the electrodeposition process. A more controlled, homogenous channel is obtained for the vertical structure. A significantly greater amount of material is needed to bridge the planar channel results, which may be seen in the bulky morphology as well as the unnecessary / unwanted material that grows surrounding the defined channel.

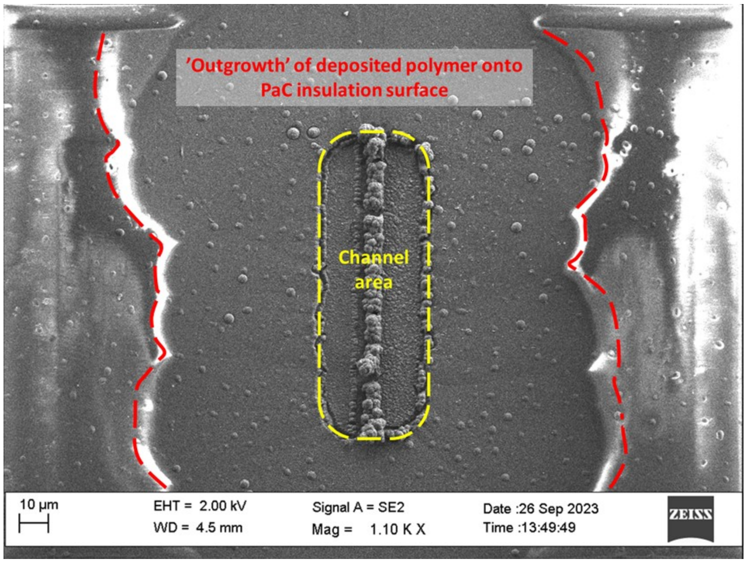

Figure S4: SEM image showing the full amount of unnecessary polymer outgrowth that results from deposition of the planar OECT channel.

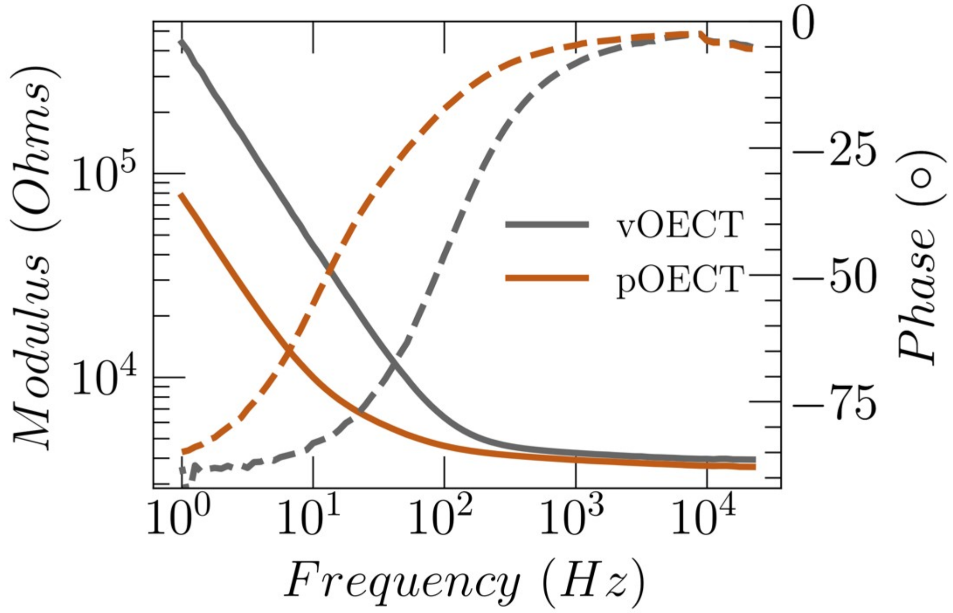

Figure S5. EIS of the pOECT (+0.28V, 240s, shorted contacts) and vOECT (Ø50 µm, 80s, shorted contacts) from Fig S1. The pOECT requires extensive polymerization time relative to the vOECT in order to bridge the contacts and form a channel. This results in more deposited material which exhibits lower impedance. Corresponding capacitance values, calculated using the model presented in Fig S6B, are 0.895 µF and 0.155 µF for the pOECT and vOECT, respectively.

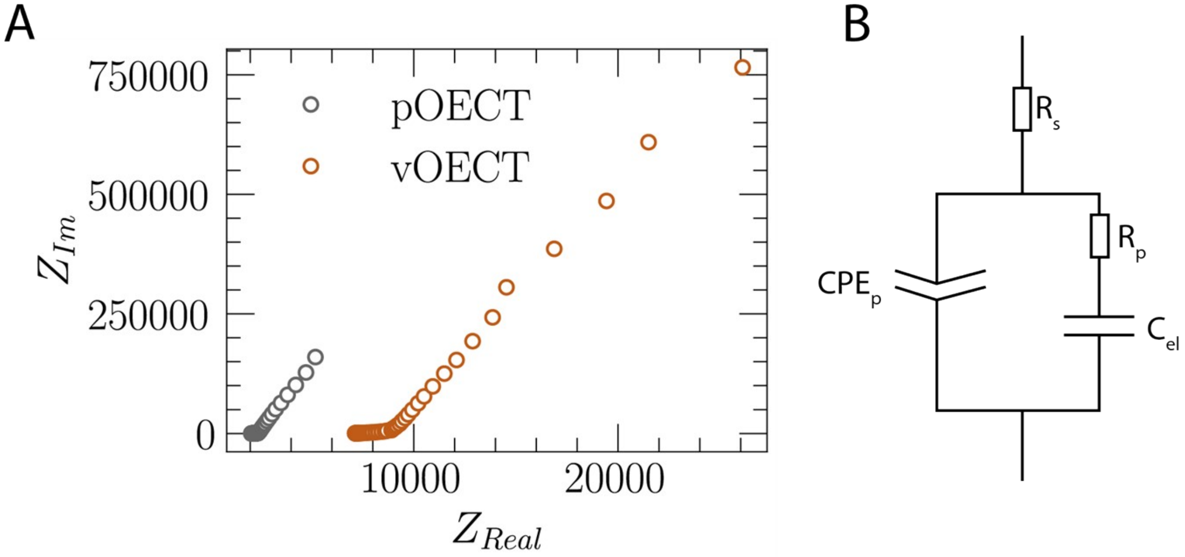

Fig S6: Electrochemical impedance spectroscopy for OECT channels grown by Method 1 – electropolymerization using a single contact. A) Nyquist plot for pOECT (grey data points) and vOECT (orange data points) and B) equivalent circuit for pOECTs and vOECTs.

Table S1. Comparison of necessary electrodeposited material to bridge the planar or vertical OECT channel. The electropolymerization charge and electrochemical capacitance values are used to demonstrate the larger amount of resulting polymer for the pOECT. Although these measurements do not give an exact quantification of the amount of material deposited, the ratio between the pOECT and vOECT is similar for both (ratio of 5.56 for the charge and 5.77 for the capacitance).

Transistor	Electropolymerization charge (μC)	Electrochemical capacitance (μF)	Error of fit parameter
pOECT (240 s)	6.06	0.895	6.74 %
vOECT (80 s)	1.09	0.155	6.09 %

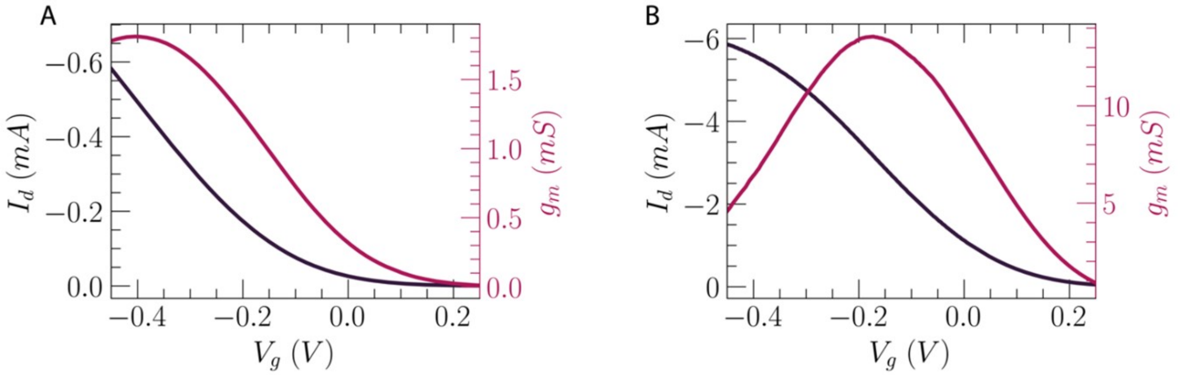

Fig S7. Transfer (black) and transconductance (red) curves for channels deposited using Method 2 - shorted contacts: A) pOECT (+0.28V, 240s), B) vOECT (+0.28V, 80s) measurement with drain connected to the top Au contact.

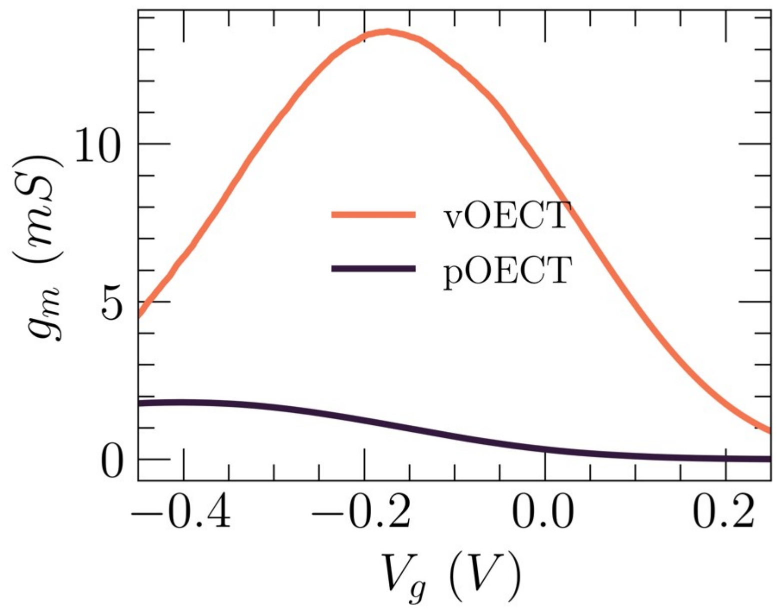

Fig S8. Comparison of transconductance curves for devices polymerized with shorted contacts, pOECT (+0.28V, 240s) and vOECT (Ø50 µm, +0.28V, 80s, measurement with drain connected to top Au contact). V_D = -0.5 V

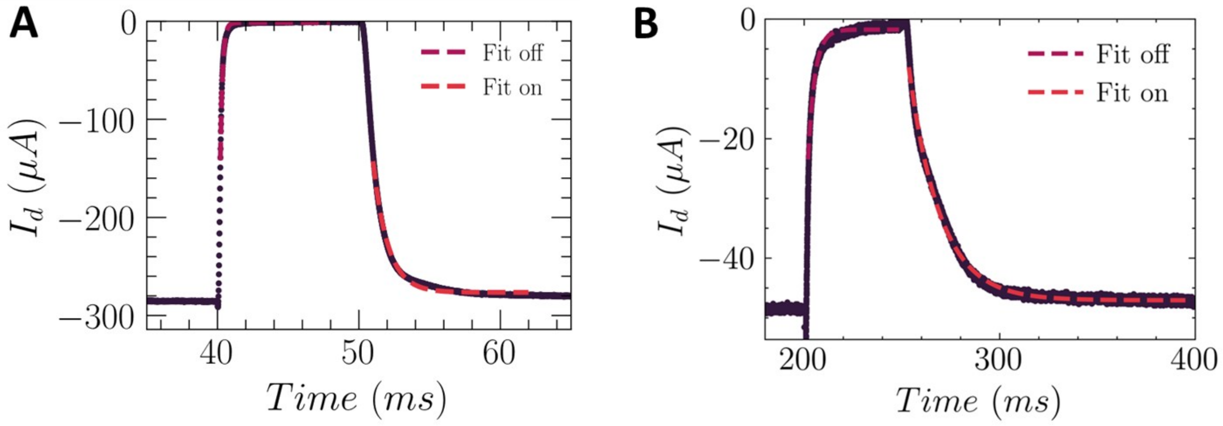

Fig S9: Response time of vOECTs (40 s, bottom) and pOECT. A) For the vOECT a short 10 ms gate voltage pulse is applied and the I_d response is recorded. The curve is fitted with an exponential model to extract the vOECT time constants: t_{off} = (1.6 e-04 ± 1.1e-06) s, t_{on} = (1.0e-03 ± 6.4e-06) s. B) For the pOECT a longer 50 ms gate pulse is used to reach the off state and the extracted time constants are: t_{off} = (3.44 e-03 ± 20e-06) s, t_{on} = (16.0e-03 ± 20e-06) s.

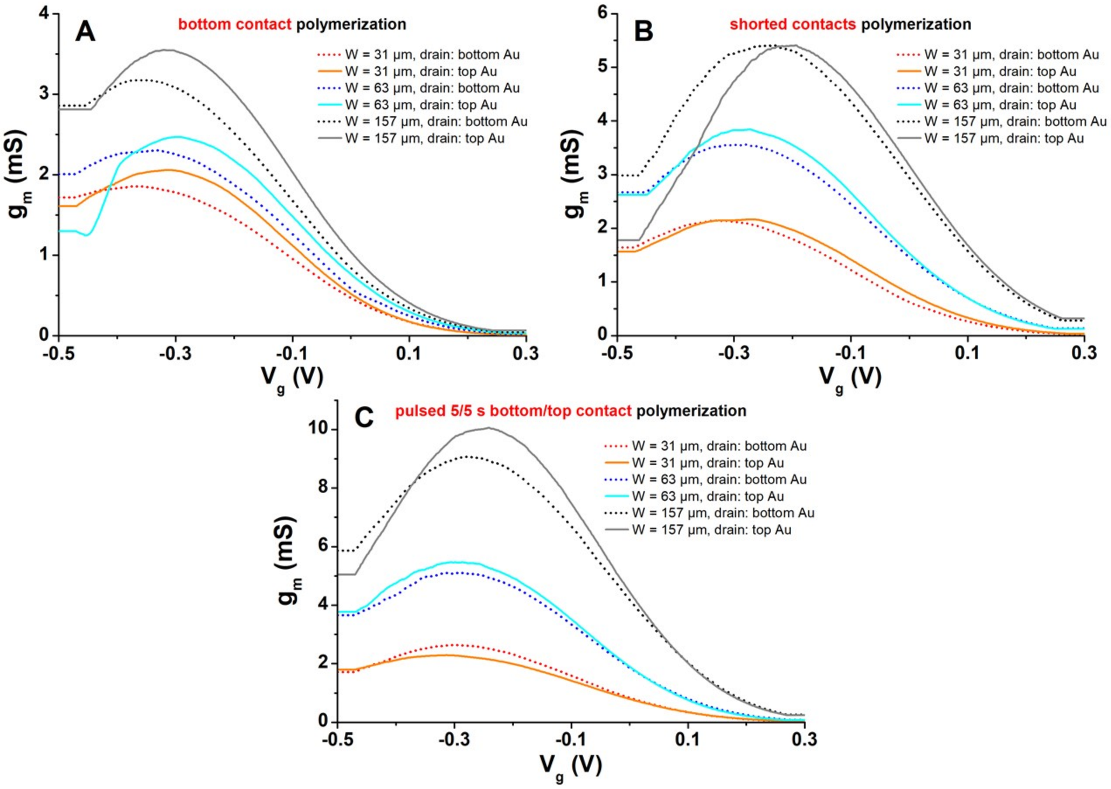

Fig S10. Transconductance curves (V_D = -0.5 V) for three different polymerization methods. A) Method 1 – polymerization from a single contact, +0.28V, 40s. B) Method 2 – polymerization with contacts shorted, +0.28V, 40s. C) Method 3 - polymerization with pulsing between top and bottom contact, +0.28V, 5 s per contact for 40 s total. Measurement made with top or bottom vOECT contact used as the transistor drain are plotted within each graph as well as geometry variation with diameter of the upper vOECT contact including 10 µm, 20 µm, and 50µm.

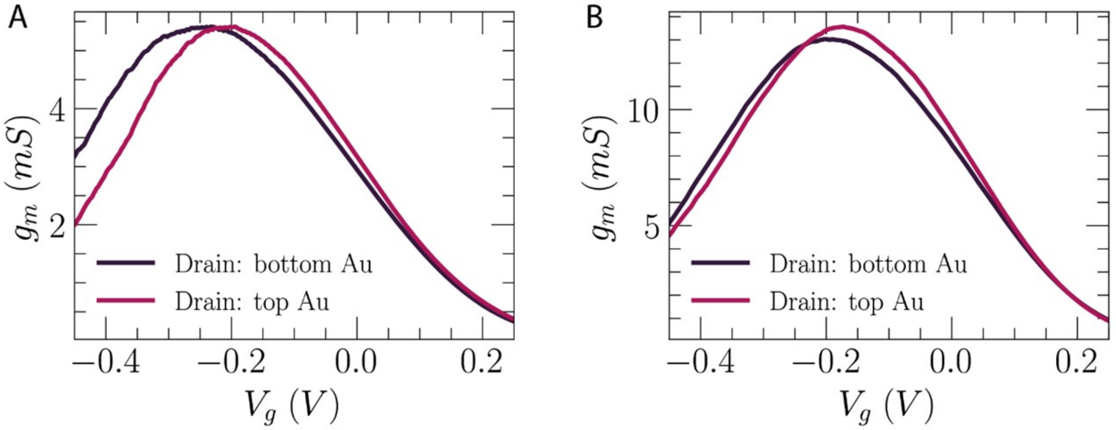

Fig S11. Transconductance curves for two different polymerization times for vOECT with deposition by Method 2. A) polymerization with shorted contacts, +0.28V, 40s B) polymerization with shorted contacts, +0.28V, 80s. Two transconductances are extracted in which drain connected either to bottom or top vOECT Au contact. V_D = -0.5 V

Table S2. Parameters of the transistors (+0.28 V, 40 s polymerization) for three different polymerization methods and transfer curves (V_D = -0.5 V) recorded with top or bottom vOECT contact as the transistor drain

Method	g_m – max (mS)	g_m – peak V_g (V)	Peak I_{DS} (mA) *For V_D = -0.5 V*
1 – Bottom electrode	BD: 3.18<br>TD: 3.55	BD: -0.362<br>TD: -0.320	BD: -1.30<br>TD: -1.45
2 – Shorted	BD: 5.41<br>TD: 5.41	BD: -0.232<br>TD: -0.195	BD: -2.54<br>TD: -2.39
3 – Pulsed	BD: 9.07<br>TD: 10.05	BD: -0.275<br>TD: -0.242	BD: -4.14<br>TD: -4.35

BD: Bottom Drain, TD: Top Drain
